# Type B aortic dissection following personalized external aortic root support (PEARS): a report of two cases

**DOI:** 10.1186/s13019-026-04120-x

**Published:** 2026-07-21

**Authors:** Alejandro González-Caldevilla Fernandez, Shivan Edmond Saith, Farhin Holia, Ana Lopez-Marco, Aung Ye Oo

**Affiliations:** 1 Division of Aortovascular Surgery, Department of Cardiothoracic Surgery, St Bartholomew’s Hospital, London, UK; 2 Queen Mary University of London, London, UK

**Keywords:** Personalized external aortic root support, PEARS, Type B aortic dissection, Heritable thoracic aortic disease, Frozen elephant trunk, Aortic surgery

## Abstract

**Background:**

Personalized external aortic root support (PEARS) is a prophylactic surgical technique designed to stabilize the aortic root in patients with heritable thoracic aortic disease (HTAD), including Marfan and Loeys-Dietz syndromes. Although early outcomes have been encouraging, distal aortic complications after PEARS remain poorly characterized. Type B aortic dissection (TBAD) following PEARS implantation has rarely been reported.

**Case presentation:**

We report two patients with HTAD who developed complicated TBAD several years after PEARS implantation. The first patient, a woman with Loeys-Dietz syndrome, presented with acute TBAD complicated by visceral malperfusion and underwent emergency total arch replacement using the frozen elephant trunk (FET) technique, followed by extensive abdominal surgery for bowel ischemia. The second patient, a man with Marfan syndrome, presented with TBAD and progressive descending thoracic aortic dilatation. He initially underwent open descending thoracic aortic replacement and subsequently required staged arch replacement with FET after retrograde extension of the dissection into the proximal aorta. Both patients survived the acute events and remain under radiological surveillance.

**Conclusions:**

Distal aortic dissection may still occur in patients with HTAD who have previously undergone PEARS implantation. Although causality cannot be inferred from isolated observations, these cases emphasize the importance of continued imaging surveillance of the entire aorta after PEARS.

## Background

Personalized external aortic root support (PEARS) is an emerging prophylactic surgical technique developed to prevent progressive dilatation of the aortic root in patients with heritable thoracic aortic disease (HTAD), particularly those with Marfan and Loeys-Dietz syndromes. By externally reinforcing the aortic root with a patient-specific macroporous mesh, the procedure aims to stabilize the ascending aorta while preserving the native aortic valve and avoiding prosthetic root replacement. Early and mid-term results have been encouraging in carefully selected patients [1, 2].

Despite these promising results, the long-term behaviour of the distal aorta following PEARS implantation remains incompletely understood. Patients with HTAD have a systemic predisposition to progressive aortic pathology, and distal aortic events have been described after other forms of proximal aortic surgery [3]. To our knowledge, detailed reports of TBAD after PEARS remain extremely limited. We therefore report two patients with HTAD who developed complicated TBAD several years after PEARS implantation, both requiring extensive surgical management.

## Case presentation

### Case 1

A 32-year-old woman with Loeys-Dietz type 2 syndrome underwent prophylactic PEARS implantation for progressive aortic root dilatation. Her past medical history included hypertension, and she was receiving beta-blocker therapy with good blood pressure control. She had no history of smoking and a positive family history of aortic disease in her father. Surveillance imaging following PEARS demonstrated stable aortic root dimensions prior to presentation.

The postoperative course was uneventful and follow-up imaging demonstrated stable proximal aortic dimensions for approximately two years.

**Fig. 1:**
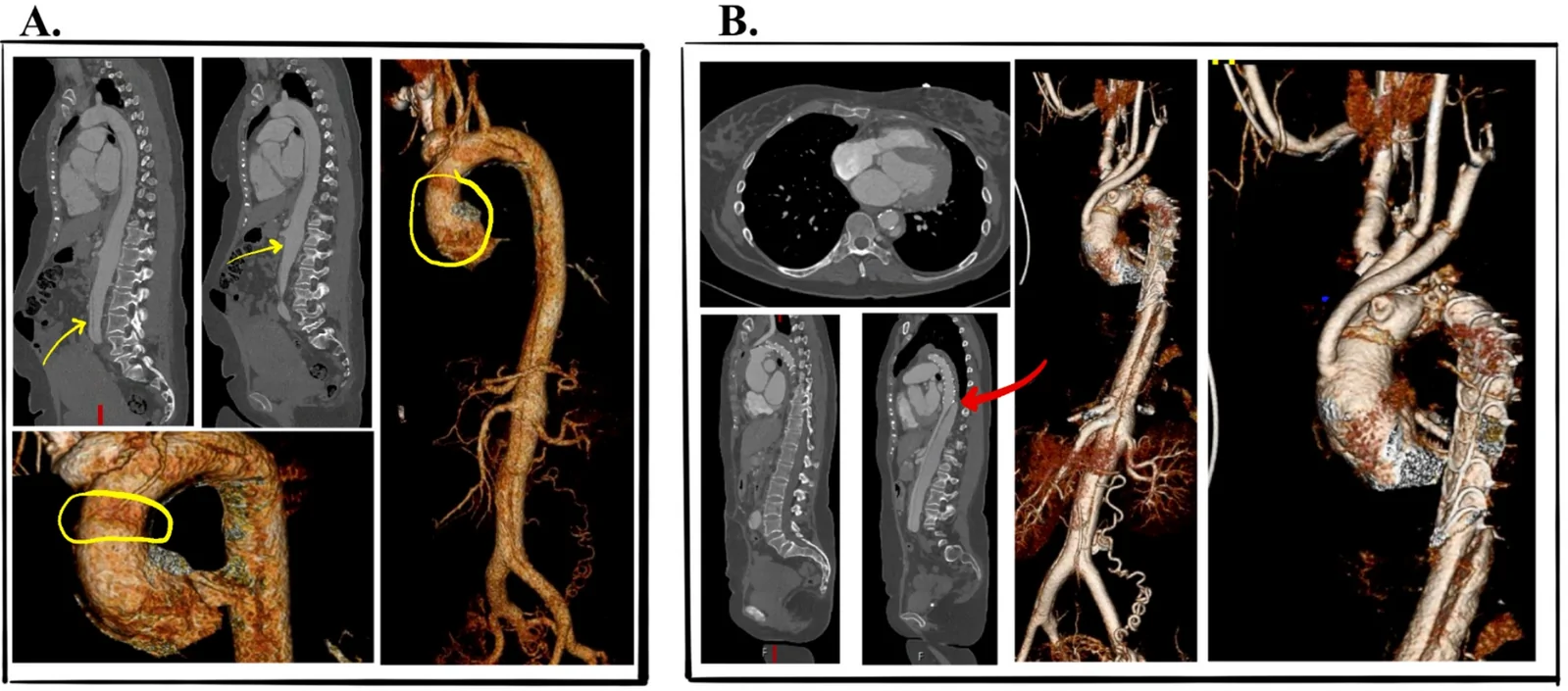
Case 1. Initial CTA demonstrating TBAD distal to the left subclavian artery with severe true lumen compression and mesenteric malperfusion; postoperative CTA after total arch replacement with FET demonstrating restoration of true lumen calibre and distal perfusion

**Fig. 2:**
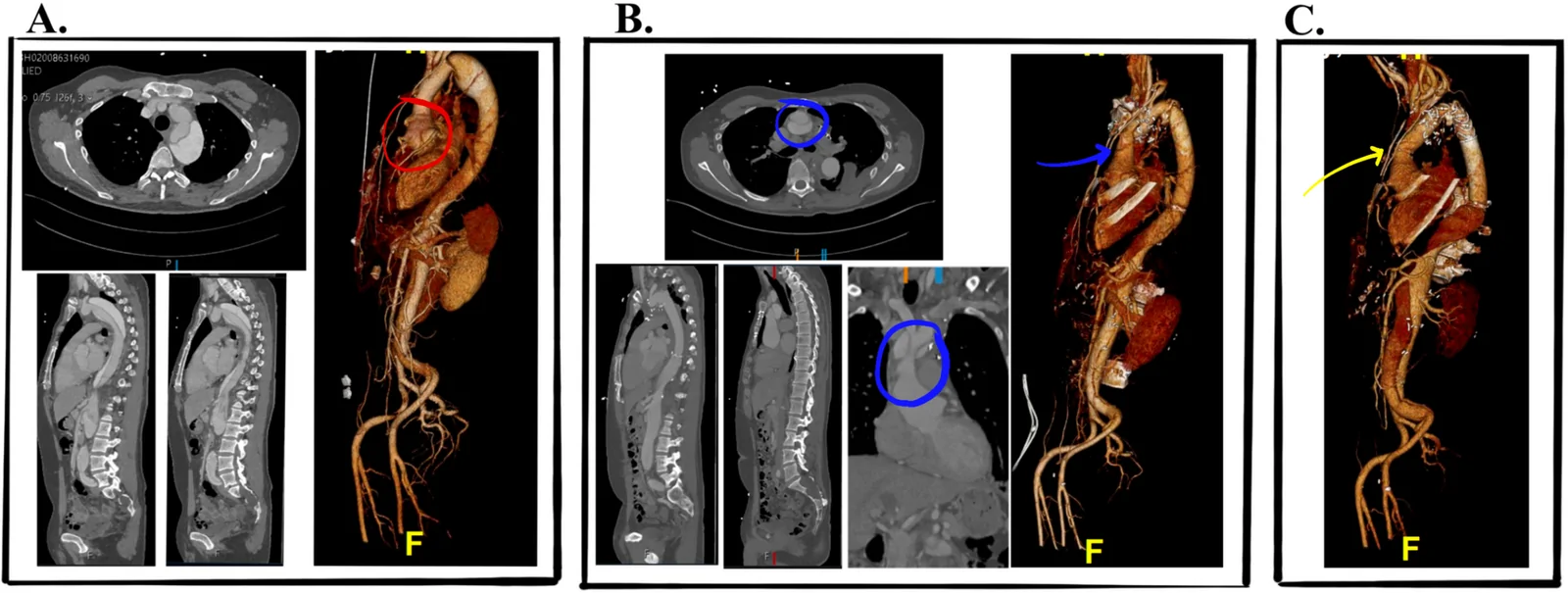
Case 2. Initial CTA showing TBAD distal to the left subclavian artery with proximal descending thoracic aortic dilatation; postoperative imaging after open descending thoracic aortic replacement showing retrograde extension into the proximal aorta; final CTA after staged total arch replacement with FET

Several years later, shortly after delivery by caesarean section, she presented with acute abdominal and lower back pain. Computed tomography angiography (CTA) demonstrated an acute TBAD with the primary entry tear distal to the left subclavian artery. The true lumen was severely compressed, with compromised visceral perfusion extending to the iliac arteries. CTA also demonstrated pneumatosis intestinalis and mesenteric venous gas, consistent with bowel ischaemia (Fig. 1). Thoracic endovascular aortic repair was considered unsuitable because of the anatomical configuration of the dissection and the degree of visceral malperfusion Fig. 2.

The patient was transferred urgently to our institution. Intraoperative findings demonstrated dissection extension involving the aortic arch. A redo sternotomy was performed and total arch replacement was undertaken from the distal ascending aorta to zone 2 using a 26 × 28 × 150 mm Thoraflex™ Hybrid prosthesis with the frozen elephant trunk technique. The proximal anastomosis was constructed at the level of the previously PEARS-supported ascending aorta, incorporating the external support within the anastomotic line.

Following completion of the aortic repair, exploratory laparotomy was performed because of persistent metabolic acidosis and radiological evidence of bowel ischaemia. Extensive intestinal necrosis was identified, requiring right hemicolectomy, extensive small-bowel resection, and cholecystectomy. Follow-up CTA demonstrated satisfactory aortic reconstruction and restoration of distal perfusion. The patient survived the acute event, was discharged home on parenteral nutrition, and remains under follow-up while awaiting bowel transplantation.

### Case 2

A 40-year-old man with Marfan syndrome confirmed by FBN1 mutation previously underwent PEARS implantation for prophylactic treatment of aortic root dilation. He was receiving beta-blocker therapy, and his blood pressure had been well controlled during follow-up. The patient had no smoking history and no known family history of aortic dissection. Surveillance imaging prior to presentation showed stable aortic root dimensions.

Several years after the index procedure, he presented with acute chest pain. CTA revealed a TBAD originating distal to the left subclavian artery, associated with proximal descending thoracic aortic dilatation measuring 50 mm. The dissection extended distally to both iliac arteries. Despite optimal medical therapy, he continued to experience pain and repeat imaging demonstrated rapid expansion of the descending thoracic aorta to 53 mm.

Given these findings, urgent open descending thoracic aortic replacement was performed via left thoracotomy using a 26 mm Gelweave™ graft under left heart bypass. The postoperative course was complicated by pneumonia and left lung atelectasis, requiring temporary re-intubation. Subsequent CTA demonstrated retrograde extension of the dissection into the proximal aorta, involving the ascending aorta within the region previously supported by PEARS.

The patient subsequently underwent redo sternotomy and replacement of the ascending aorta and arch using the frozen elephant trunk technique with a 26 × 28 × 100 mm Thoraflex™ Hybrid prosthesis. The proximal anastomosis was again constructed at the level of the PEARS-supported ascending aorta. He recovered well and was discharged home. Follow-up imaging demonstrated progressive dilatation of the abdominal visceral aorta, which remains under evaluation for further surgical management Table 1.

**Table 1:** Clinical characteristics of the two reported cases

Variable	Case 1	Case 2
Age at TBAD	32 years	40 years
Underlying condition	Loeys-Dietz syndrome	Marfan syndrome
Gene/variant	TGFBR2	FBN1
Time from PEARS to TBAD	Approximately 2 years	Approximately 4 years
Entry tear location	Distal to left subclavian artery	Distal to left subclavian artery
Extent of dissection	Arch to iliac arteries with visceral malperfusion	Descending thoracic aorta to iliac arteries
Initial intervention	Total arch replacement with FET	Open descending thoracic aortic replacement
Subsequent intervention	Emergency abdominal surgery	Staged arch replacement with FET
Current status	Alive; awaiting bowel transplantation	Alive; abdominal aorta under evaluation

## Discussion and conclusions

PEARS has emerged as an alternative prophylactic strategy for selected patients with HTAD, particularly those with early aortic root dilatation and preserved aortic valve function. By externally reinforcing the aortic root, the procedure aims to prevent progressive dilatation while preserving native valve anatomy [1, 2]. However, its long-term effect on the downstream aorta remains uncertain.

One possible mechanism for distal aortic events after PEARS is compliance mismatch between the externally supported ascending aorta and the untreated distal thoracic aorta. Reinforcement of the proximal aorta may alter segmental distensibility, pulse-wave transmission, and downstream wall stress. A second hypothesis relates to the transition zone at the distal edge of the supported segment, where local stress concentration may occur in an intrinsically fragile aorta. Similar interface phenomena have been discussed after other forms of proximal aortic surgery [3, 4]. At the same time, distal dissection after PEARS may simply reflect the natural history of diffuse HTAD rather than a direct consequence of the procedure itself.

The educational value of these cases lies in the fact that both patients developed severe distal aortic complications after apparently successful prophylactic root stabilisation and required complex rescue surgery. These observations support lifelong surveillance of the entire thoracic and abdominal aorta after PEARS. In clinical practice, annual CTA or magnetic resonance angiography appears reasonable, with shorter intervals if distal dilatation, symptoms, or interval growth are detected.

In conclusion, TBAD may still occur in patients with HTAD who have previously undergone PEARS implantation. Although a causal relationship cannot be established from two cases, these observations highlight the importance of whole-aorta surveillance and careful interpretation of downstream aortic risk in this population.

## Data Availability

All data generated or analysed during this study are included in this published article. Additional anonymised details are available from the corresponding author on reasonable request, subject to institutional approvals.

## Abbreviations

CTA: Computed tomography angiography
FET: Frozen elephant trunk
HTAD: Heritable thoracic aortic disease
PEARS: Personalized external aortic root support
TBAD: Type B aortic dissection

## References

[1] Pepper J, Golesworthy T, Austin C, et al. Personalized external aortic root support (PEARS): a narrative review. J Thorac Cardiovasc Surg. 2024;168(6):1628–31. 10.1016/j.jtcvs.2024.07.016. 39009338

[2] Van Hoof L, Lamberigts M, Noé D, et al. Matched comparison between external aortic root support and valve-sparing root replacement. Heart. 2023;109(10):832–8. 10.1136/heartjnl-2022-321840. PMC10313978 36650042

[3] Zhou X, Wang X, Li J, et al. Fate of the distal aorta following root replacement in Marfan syndrome. Front Cardiovasc Med. 2023;10:1186181. 10.3389/fcvm.2023.1186181. PMC10338094 37448791

[4] Zakko J, Ghincea C, Reece TB. Future paradigms of aortic dissection. Cardiol Clin. 2024;42(4):569–79. 10.1016/j.ccl.2024.12.001. 40268361

[5] Hameed I, Cifu AS, Vallabhajosyula P. Management of thoracic aortic dissection. JAMA. 2023;329(9):756–7. 10.1001/jama.2023.0265. 36795378

